# Complete genome sequence of the archetype bile acid 7α-dehydroxylating bacterium, *Clostridium scindens* VPI12708, isolated from human feces, circa 1980

**DOI:** 10.1128/MRA.00029-23

**Published:** 2023-08-09

**Authors:** Kelly Yovani Olivos Caicedo, Francelys V. Fernandez-Materan, Alvaro G. Hernandez, Steven L. Daniel, Joao M. P. Alves, Jason M. Ridlon

**Affiliations:** 1 Department of Parasitology, Institute of Biomedical Sciences, University of São Paulo, São Paulo, Brazil; 2 Carl R. Woese Institute for Genomic Biology, University of Illinois Urbana-Champaign, Urbana, Illinois, USA; 3 Department of Animal Sciences, University of Illinois Urbana-Champaign, Urbana, Illinois, USA; 4 Keck Center for Biotechnology, University of Illinois Urbana-Champaign, Urbana, Illinois, USA; 5 Department of Biological Sciences, Eastern Illinois University, Charleston, Illinois, USA; 6 Division of Nutritional Sciences, University of Illinois Urbana-Champaign, Urbana, Illinois, USA; 7 Cancer Center at Illinois, Urbana, Illinois, USA; 8 Center for Advanced Study, University of Illinois Urbana-Champaign, Urbana, Illinois, USA; 9 Department of Microbiology and Immunology, Virginia Commonwealth University, Richmond, Virginia, USA; University of Rochester School of Medicine and Dentistry, Rochester, New York, USA

**Keywords:** *Clostridium scindens*, 7α-dehydroxylating bacteria, genome sequencing, genome assembly, bile acid

## Abstract

*Clostridium scindens* strain VPI12708 serves as model organism to study bile acid 7α-dehydroxylating pathways. The closed circular genome of *C. scindens* VPI12708 was obtained by PacBio sequencing. The genome is composed of 3,983,052 bp, with 47.59% G + C, and 3,707 coding DNA sequences are predicted.

## ANNOUNCEMENT

Originally isolated from a fecal sample of a colon cancer patient in 1980 and described as *Eubacterium* strain sp. VPI12708 (1, 2), later reclassified as *Clostridium scindens* VPI12708 (3), and most recently as “*Lachnoclostridium scindens”* (4). This strain encodes several enzymes involved in bile acid 7α-dehydroxylation, standing out as a model organism from which these genes were cloned (5 - 9) and the biochemical intermediates in bile acid metabolism elucidated (10 - 12).

The donated *C. scindens* VPI12708 strain was reconstituted from cryostock culture from clinical fecal samples storage at USDA-ARS-NCAUR in 30% glycerol and −80°C temperature. Initially cultured in anaerobic trypticase soy broth (TSB) (2), the strain was diluted and plated on TSB with 10% sheep’s blood. Uniform colonies were observed, and characteristic phenotypes of this strain were confirmed (13, 14). Genomic DNA was isolated as previously described (15). DNA concentration of 46.8 ng·μL^−1^ (*A*_260 nm_/*A*_280 nm_ = 1.87) was determined by Nanodrop, and two high molecular weight bands were observed above the 12 kb band of a 1-kB DNA marker (Quick-Load 1 kB DNA Ladder, NEB) on a 0.5% 1× TAE gel stained with GelRed.

The genomic DNA was sheared with a Megaruptor 3 to an average fragment length of 13 kb. Sheared gDNA was converted to a library with the SMRTBell Express Template Prep Kit 3.0 at the Roy J. Carver Biotechnology Center, University of Illinois at Urbana-Champaign. The library was sequenced on one SMRT Cell 8M on a PacBio Sequel IIe using the circular consensus sequencing (CCS) mode and a 30-h movie time. CCS analysis was done in instrument with SMRTLink V11.0 using the following parameters: --ccs --min-passes 3 --min-rq 0.99.

PacBio generated a total of 310,426 reads with a minimum and maximum read length (bp) of 34,778 and 1,246, respectively. A mean read length of 9,743 bp (N50 contig length of 10,501 bp) was obtained, and PacBio reads were subjected to quality control using the FastQC v0.11.9 tool (16). Reads with less than 10K bases in length were removed using cutadapt v4.0 (17). Subsequently, to lower the coverage to around 50-fold, 17,000 of the remaining reads were randomly collected with seqtk v.1.2-r94 (https://github.com/lh3/seqtk). The 17,000 selected reads used for genome assembly presented a mean read length of 12,564 bp and an N50 contig length of 12,420 bp.

Genome assembly was performed with the Canu v.2.2 assembler (18). To assess genome assembly completeness, BUSCO v.5.3.1 (19) was used. A single circular contig assembly was generated with an overall assembly containing 3,983,052 bp and 47.59% G + C content. Annotation was performed with the rapid prokaryotic genome annotation tool Prokka v.1.14.5 (20). Default parameters were used for all software. Approximately 91.78% of the genome is composed of predicted genes, with 3,707 coding DNA sequences, 12 ribosomal RNA genes, 56 transfer RNA genes, 1 tmRNA gene, and 1 pseudogene.

## Data Availability

This whole genome shotgun project has been deposited in GenBank under the accession no. CP113781; project data are available under BioProject accession number PRJNA902789, with BioSample accession number SAMN31775693 and SRA accession number SRR23686740.

## References

[1] Hylemon PB , Cacciapuoti AF , White BA , Whitehead TR , Fricke RJ . 1980. 7α-Dehydroxylation of cholic acid by cell extracts of Eubacterium species VPI 12708. Am J Clin Nutr 33:2507–2510. doi:10.1093/ajcn/33.11.2507 7435421

[2] White BA , Lipsky RL , Fricke RJ , Hylemon PB . 1980. Bile acid induction specificity of 7 alpha-dehydroxylase activity in an intestinal Eubacterium species. Steroids 35:103–109. doi:10.1016/0039-128x(80)90115-4 7376208

[3] Kitahara M , Takamine F , Imamura T , Benno Y . 2000. Assignment of Eubacterium sp. VPI 12708 and related strains with high bile acid 7alpha-dehydroxylating activity to Clostridium scindens and proposal of Clostridium hylemonae sp. nov., isolated from human faeces. Int J Syst Evol Microbiol 50 Pt 3:971–978. doi:10.1099/00207713-50-3-971 10843034

[4] Adams SH , Wright R , Piccolo BD , Moody B , Sikes J , Avaritt N , Chintapalli SV , Ou X . 2022. C-section increases cecal abundance of the archetypal bile acid and glucocorticoid modifying Lachnoclostridium [Clostridium] scindens in mice. Physiol Rep 10:e15363. doi:10.14814/phy2.15363 35778808 PMC9249977

[5] White BA , Cacciapuoti AF , Fricke RJ , Whitehead TR , Mosbach EH , Hylemon PB . 1981. Cofactor requiremets for 7 alpha-dehydroxylation of cholic and chenodeoxycholic acid in cell extracts of the intestinal anaerobic bacterium, Eubacterium species V.P.I. 13708. J Lipid Res 22:891–898. 7276750

[6] Mallonee DH , White WB , Hylemon PB . 1990. Cloning and sequencing of a bile acid-inducible operon from Eubacterium sp. strain VPI 12708. J Bacteriol 172:7011–7019. doi:10.1128/jb.172.12.7011-7019.1990 2254270 PMC210822

[7] Gopal-Srivastava R , Mallonee DH , White WB , Hylemon PB . 1990. Multiple copies of a bile acid-inducible gene in Eubacterium sp. strain VPI 12708. J Bacteriol 172:4420–4426. doi:10.1128/jb.172.8.4420-4426.1990 2376563 PMC213270

[8] Coleman JP , White WB , Lijewski M , Hylemon PB . 1988. Nucleotide sequence and regulation of a gene involved in bile acid 7-dehydroxylation by Eubacterium sp. strain VPI 12708. J Bacteriol 170:2070–2077. doi:10.1128/jb.170.5.2070-2077.1988 2834320 PMC211088

[9] Coleman JP , White WB , Hylemon PB . 1987. Molecular cloning of bile acid 7-dehydroxylase from Eubacterium sp. strain VPI 12708. J Bacteriol 169:1516–1521. doi:10.1128/jb.169.4.1516-1521.1987 3549693 PMC211977

[10] Hylemon PB , Melone PD , Franklund CV , Lund E , Björkhem I . 1991. Mechanism of intestinal 7 alpha-dehydroxylation of cholic acid: evidence that allo-deoxycholic acid is an inducible side-product. J Lipid Res 32:89–96. 2010697

[11] Björkhem I , Einarsson K , Melone P , Hylemon P . 1989. Mechanism of intestinal formation of deoxycholic acid from cholic acid in humans: evidence for a 3-oxo-delta 4-steroid intermediate. J Lipid Res 30:1033–1039. 2794786

[12] Lee JW , Cowley ES , Wolf PG , Doden HL , Murai T , Caicedo KYO , Ly LK , Sun F , Takei H , Nittono H , Daniel SL , Cann I , Gaskins HR , Anantharaman K , Alves JMP , Ridlon JM . 2022. Formation of secondary allo-bile acids by novel enzymes from gut Firmicutes. Gut Microbes 14:2132903. doi:10.1080/19490976.2022.2132903 36343662 PMC9645264

[13] Ridlon JM , Kang DJ , Hylemon PB . 2010. Isolation and characterization of a bile acid inducible 7alpha-Dehydroxylating Operon in Clostridium hylemonae TN271. Anaerobe 16:137–146. doi:10.1016/j.anaerobe.2009.05.004 19464381 PMC6262846

[14] de Prada P , Setchell KD , Hylemon PB . 1994. Purification and characterization of a novel 17 alpha-hydroxysteroid dehydrogenase from an intestinal Eubacterium sp. VPI 12708. J Lipid Res 35:922–929. 8071614

[15] Ridlon JM , McGarr SE , Hylemon PB . 2005. Development of methods for the detection and quantification of 7alpha-dehydroxylating clostridia, Desulfovibrio vulgaris, Methanobrevibacter smithii, and Lactobacillus plantarum in human feces. Clin Chim Acta 357:55–64. doi:10.1016/j.cccn.2005.02.004 15963794

[16] Andrews, S. (2010). FastQC: a quality control tool for high throughput sequence data http://www.bioinformatics.babraham.ac.uk/projects/fastqc/

[17] Martin M . 2011. Cutadapt removes adapter sequences from high-throughput sequencing reads. EMBnet j 17:10. doi:10.14806/ej.17.1.200

[18] Koren S , Walenz BP , Berlin K , Miller JR , Bergman NH , Phillippy AM . 2017. Canu: scalable and accurate long-read assembly via adaptive k-mer weighting and repeat separation. Genome Res 27:722–736. doi:10.1101/gr.215087.116 28298431 PMC5411767

[19] Simão FA , Waterhouse RM , Ioannidis P , Kriventseva EV , Zdobnov EM . 2015. BUSCO: assessing genome assembly and annotation completeness with single-copy orthologs. Bioinformatics 31:3210–3212. doi:10.1093/bioinformatics/btv351 26059717

[20] Seemann T . 2014. Prokka: rapid prokaryotic genome annotation. Bioinformatics 30:2068–2069. doi:10.1093/bioinformatics/btu153 24642063

